# Epidemiological Profile of Patients with Cutaneous Melanoma in a Region of Southern Brazil

**DOI:** 10.1155/2012/917346

**Published:** 2012-03-27

**Authors:** Marcelo Moreno, Ricardo Ludwig Schmitt, Maria Gabriela Lang, Vanessa Gheno

**Affiliations:** Universidade Comunitária da Região de Chapecó-Unochapecó, Avenida Senador Atílio Fontana, 591-E. Efapi, Caixa Postal 1141 89809-000, Chapecó, SC, Brazil

## Abstract

Cutaneous melanoma (CM) is responsible for 75% of deaths from malignant skin cancer. The incidence of CM in the southern region of Brazil, particularly in the western region of Santa Catarina, is possibly higher than estimated. In this study, the clinical and epidemiological profile of patients with CM treated in the western region of Santa Catarina was examined. A cross-sectional study was performed with patients diagnosed with CM from January 2002 to December 2009, from 78 counties of the western region of the state of Santa Catarina. Data were collected using a protocol adapted from the Brazilian Melanoma Group and 503 patients were evaluated. The incidence and prevalence of CM found in this region are much higher than those found elsewhere in the country. This fact is most likely due to the phenotypic characteristics of the population and the high incidence of UV radiation in this region due to its location in southern Brazil, as is the case in the countries of Oceania.

## 1. Introduction

Among skin cancers, cutaneous melanoma (CM) is the least frequent. However, it presents a higher mortality rate, mostly due to its ability to invade and spread through different tissues [1]. Nevertheless, when detected in its early stages, it has a high cure rate [2].

CM occurs predominantly in white populations [1]. The CM incidence rates are particularly high in Northern Europe, North America, and Australia and low among the indigenous population of Africa, Asia, Latin America, and southern Europe [3]. In New Zealand and Australia, in 2002, a global analysis showed an incidence of CM among men of 37.7 cases per 100 000 inhabitants and 29.4 among women [4].

Queensland, Australia, is regarded as having the highest incidence of melanoma worldwide: 51 per 100 000 in 2002 [5]. In the region of Tauranga in New Zealand during the year of 2003, an incidence of 70 per 100 000 inhabitants, even higher than the Australian statistics, was reported [6]. One explanation would be that the incidence of CM is also strongly correlated with the latitude of the region, being higher in areas of high solar radiation [5, 7].

Studies related to the incidence of melanoma in Brazil are still scarce. According to the Brazilian National Cancer Institute (INCA), the national incidence rate estimated for 2012 is low: 3170 new cases in men and 3060 new cases in women. The highest estimated rates are in the south. However, due to population characteristics and geographic localization, the incidence of CM in southern Brazil is possibly higher than estimated [8]. Geographically, Australia and New Zealand (Queensland—26° to 29°S) and southern Brazil (western Santa Catarina—26° to 27°S) are at similar latitudes [9, 10].

In Brazil, compulsory notification of cancer cases is not yet a reality nationwide, occurring in only 30% of the country. For this reason, the existence of a considerable number of subrecords is possible [8].

The present study aimed to delineate the epidemiological profile of patients with CM treated in the western region of the state of Santa Catarina, Brazil.

## 2. Materials and Methods

A cross-sectional study was performed combining research during the period from 2002 to 2009, with longitudinal research during the year 2009. The study was reviewed and approved by the Ethics Research Committee of Unochapecó, Santa Catarina, Brazil. Data were collected from the regional cancer notification service and from medical records of the referral service for cancer treatment, using as reference the modified protocol of the Brazilian Melanoma Group [11]. The incomplete data were filled in by contacting patients, family, physicians, and health services by telephone. All records pertaining to the western region of Santa Catarina (Figure 2) with an initial diagnosis of CM were included in this study and excluded those with ocular melanoma, mucosal melanoma, and visceral melanoma. Demographic data were extracted from the healthcare database of the Brazilian government (DATASUS) [12]. Statistical analysis was performed using the software SPSS *Statistics *version 19.0. For comparison of continuous variables, the Student's *t*-test was used, while the Chi-square and Fisher's test were used for nominal variables.

## 3. Results

### 3.1. Epidemiology of CM in the Western Region of Santa Catarina

In eight years, 503 cases were registered, but 6 were excluded from the study due to lack of data. The number of CM cases per year in the region ranged from 50 in 2002 up to 89 in 2008, resulting in an incidence of 7.4 and 12.2 per 100 000 inhabitants, respectively. The prevalence of CM in the region over a period of eight years was 71.5 cases per 100 000 inhabitants, and the incidence in 2009 was 9.3 cases per 100 000 inhabitants (Table 1).

Patients were from 78 different counties. Most were originally from Chapecó (26.2%) followed by São Miguel do Oeste (5.2%). The county with the highest prevalence of CM during this period was São João do Oeste, with 305.6 cases per 100 000 inhabitants. The county of the greatest incidence of CM in 2009 was Nova Itaberaba, with 71.1 cases per 100,000.

**Figure 1:**
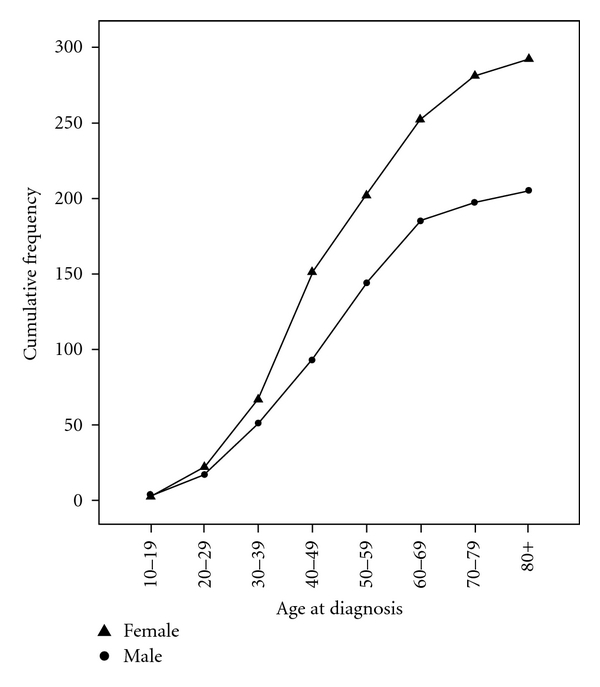
Cumulative frequency distribution of age-specific rates for cutaneous melanoma in the western region of Santa Catarina, Brazil, by gender from 2002 to 2009 (xy men and xy women).

**Figure 2:**
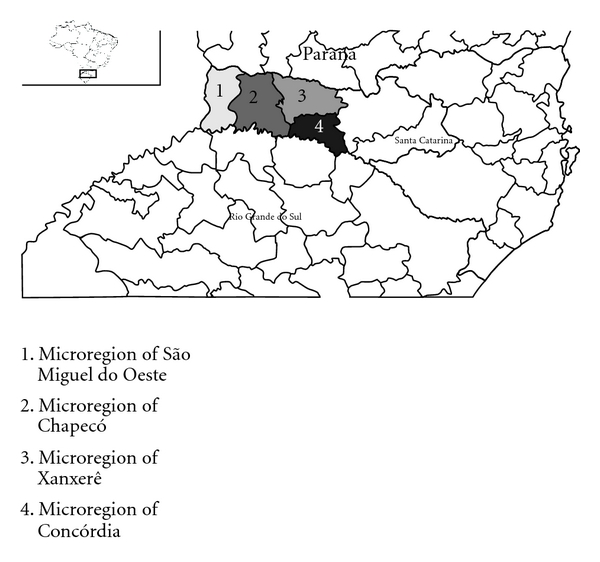
Western region of the state of Santa Catarina, Brazil, from the Brazilian Institute of Geography and Statistics, 2010.

As shown in Figure 1, a higher CM prevalence was found among individuals older than 50 years, with the highest rate being found among those aged 80 years or more. However, this age group represents the minority of patients in the region.

### 3.2. Clinical Characteristics of Patients

The average patient age was 50.6 years (SD = 15.4 years), with ages ranging from 14 to 90 years. Most patients were in the economically active age group (20 to 50 years), which accounted for 50.7% of individuals.

We found that the disease is more prevalent among women (292 cases) than among men (205 cases) (*P* < 0.01). The incidence in 2009 was 10.21 per 100 000 women and 8.5 per 100 000 men, while the prevalence over a period of eight years was 84.2 per 100 000 women and 58.8 per 100 000 men. The average age among females was 50.8 years (SD = 15.6) and 50.4 years for males (SD = 15.2) (Figure 1).

A total of 445 (92.7%) patients were skin types I and II. A total of 237 (52.5%) had light hair and 247 (54.8%) had light-colored eyes (green or blue). Other characteristics evaluated were the history of ephelides in childhood, which was positive in 166 cases. Occupational sun exposure was considered high in 327 cases. Thirty-eight patients had a history of sunburn during childhood and 73 during adulthood. A total of 167 presented sunburn history in both childhood and adulthood; 164 presented no history of sunburn. The presence of congenital nevus related to melanoma was positive in 84 cases. One hundred forty-three patients presented multiple nevi. Of these, only 51 presented more than 50 nevi. The presence of atypical nevi detected by histopathological examination occurred in 43 cases.

**Figure 3:**
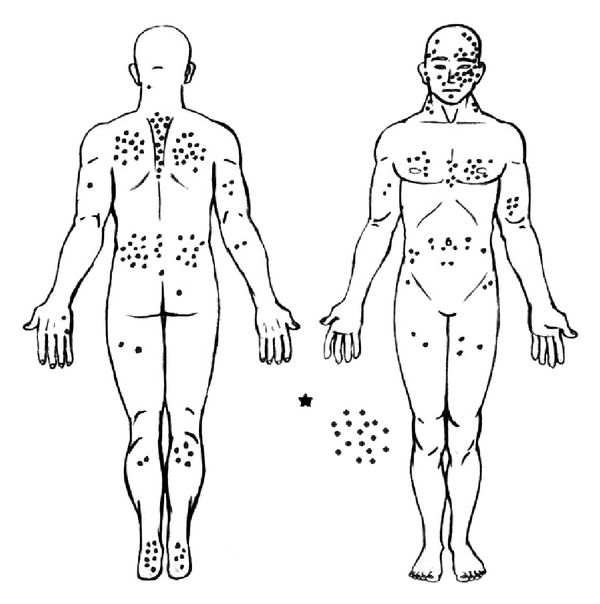
Anatomical sites of cutaneous melanoma lesions in male patients in the western region of Santa Catarina, Brazil, from 2002 to 2009 (*n* = 205). *Patients with metastatic cutaneous melanoma for lymph nodes and unknown primary cutaneous site on first presentation.

**Figure 4:**
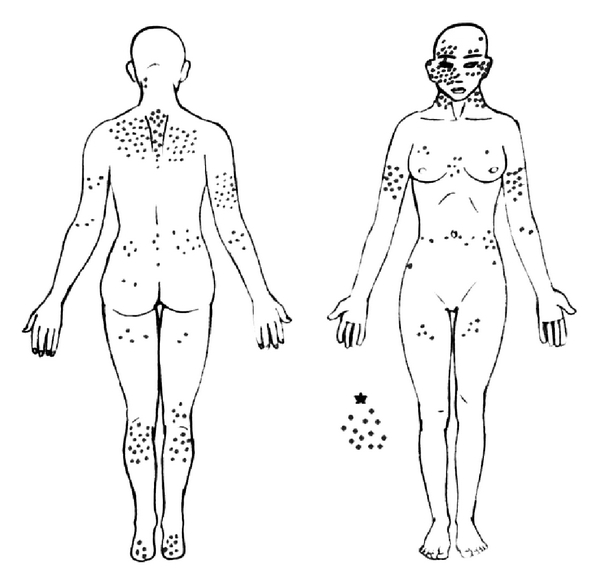
Anatomical sites of cutaneous melanoma lesions in women patients in western region of Santa Catarina, Brazil, from 2002 to 2009 (*n* = 292). *Patients with metastatic CM for lymph nodes and unknown primary cutaneous site on first presentation.

Men showed a predominance of CM occurring on the head, neck, and trunk areas (58.9%) and women on the limbs (59.8%) (Figures 3, 4 and Table 2).

### 3.3. Histopathological Characteristics of Patients

The skin lesions presented by the patients underwent histopathological evaluation by specialized laboratories which followed the standards recommended by the Brazilian Melanoma Group (BMG). Regarding the size (area in mm²) of the primary lesion, there was a variation between 1.04 and 9.090 with an average of 419.9 (SD = 972.8). The lesions of 25 patients were not measured, and 28 had metastatic disease of unknown primary site at the time of diagnosis.

We found that the most common histological type was superficial spreading CM (SSM) with 287 cases (59.92%), followed by nodular CM (NM) with 90 cases (18.79%), lentigo maligna CM (LMM) with 33 (8.89%), acral lentiginous (ALM) with 17 (3.55%), and desmoplastic (DM) in 3 cases (0.63%).

When considering the sex of the patients, differences were observed in relation to the variability in pathological staging. Cutaneous lesions, classified as pTis, pT1, and pT2, were more common in females when compared to males (*P* = 0.01). Ulcerations were more common among men (41.6%) than among women (30.3%) (*P* = 0.01).

The average mitotic rate was 3.4 mitoses per mm² (SD = 5.04). Considering the invasion of adjacent structures, vascular or lymphatic invasion was observed in 40 patients and perineural invasion was present in 10 patients. Peritumoral inflammatory infiltrate appeared in 287 lesions and intratumoral in 222.

Of the 45.3% of patients who were submitted to sentinel lymph node research, 16.4% were SLN positive and underwent radical lymphadenectomy. Of these, 14.5% showed other lymph node metastases. In addition to SLN-positive patients, 72 also underwent radical lymphadenectomy due to clinically compromised lymph nodes (Table 2).

Upon clinical staging at the moment of diagnosis (*n* = 464), it was found that most patients analyzed (39.4%) were in stage I. A total of 102 individuals (21.9%) were in stage II, 72 (15.5%) were in stage III, and 52 (11.2%) already had advanced disease and were classified as stage IV.

## 4. Discussion

### 4.1. Epidemiology

Studies have reported a sharp increase in incidence and mortality of CM among the Caucasian population in the last century, which represents an important public health problem [4, 13–15]. The first step towards developing strategies for disease prevention is to evaluate the magnitude of the problem and the characteristics of the affected population [13].

To standardize the epidemiological data related to melanoma, in 2004 the BMG established the Registro Brasileiro de Melanoma (Brazilian Melanoma Registry) which enables complete clinical assessment of patients and subsequent dissemination of national data [11].

The city of Chapecó is the official registration center of CM cases in the western region of Santa Catarina for the treatment of this malignancy. A previous study assessing the western region of Santa Catarina, carried out by Moreno (2005) in the period between 2002 and 2005, reported an average incidence of 14.5 cases per 100 000 inhabitants (data not published). In this study, the prevalence of CM in the region, over a period of eight years, was 71.5 cases per 100 000 inhabitants and in 2009 the incidence was 9.3 cases per 100 000 inhabitants. The town of Nova Itaberaba had the highest incidence rate, 71.1 cases per 100 000 inhabitants. These data demonstrate that the occurrence of this disease, in this region of Brazil, is much higher than national and global levels.

### 4.2. Geographic Location

In 1956, Lancaster suggested that the geographical distribution of mortality from CM was related to increased sun exposure in areas with low latitude, such as New Zealand and Australia, which have the highest rates of CM in the world [16, 17]. More recently, another study conducted in Europe reaffirmed the hypothesis that locations closer to the equator are associated to higher incidence rates of CM [18].

Brazil, the largest country in South America and the world's fifth largest country by geographical area, is located between latitudes 5°N and 34°S. Due to this characteristic, different climates exist throughout the country [10]. A recent work failed to characterize the existence of seasonal variability of CM in the state of Rio Grande do Sul, but found an incidence well above that seen in the rest of the country, suggesting that this is mainly due to low latitude [19]. The state of Santa Catarina is located between parallel 25° and 29°S, similar to the Australia and New Zealand location (10° and 39°S) [9, 10]. This could be an explanation for the high rates of CM in the region, added to the fact that the population is composed mostly of Caucasians.

### 4.3. UV Radiation and Ethnicity

After 50 years of epidemiological research, Eide and Weinstock [2] found that there is a low incidence of CM among Hispanics, Asians, and blacks. This has been attributed to the protective effect of skin with dark pigmentation [2]. An American study, which evaluated data from 11 centers for cancer registries over a period of 10 years, pointed to UV radiation as a key risk factor in the etiology of CM, especially in individuals with skin types I and II [20]. The population of western Santa Catarina has a higher risk of CM since it is an area colonized primarily by Caucasians. In this study we found that 99.6% of patients were white and, among them, 92.7% had skin types I and II. The presence of ephelides was observed in 38% of subjects, showing poor solar tolerance [2, 21].

Sun exposure, an important risk factor for developing CM, is even more damaging when it occurs in childhood in an intense and intermittent way [1, 22]. Many of the patients analyzed in this work presented the following risks: 66.6% worked in places of high sun exposure and 54.1% had a history of sunburn during childhood.

Besides the fact that the incidence of UV radiation increases by 7% during the summer in the southern hemisphere, if compared to the northern hemisphere [23], the southern region presents a smaller thickness of the ozone layer along with a lower amount of dust and pollutants than the northern hemisphere, which facilitates the penetration of UV rays [23] According to statistics from the National Institute for Space Research (INPE), the incidence of UV on a typical summer day varied between 8 and 12 in western Santa Catarina. This level of radiation is very high for human populations, requiring extreme protective measures [24].

### 4.4. Gender and Age

In agreement with previous studies [25, 26], we observed a higher incidence of CM in women (58.75%). The average age of CM patients in the western region of Santa Catarina was approximately 50 years, similar to that reported in a study performed with 365 patients diagnosed with CM in the city of São Paulo, Brazil, from May 1993 to January 2006 [14]. Nevertheless, in support of findings that demonstrate an increase in prevalence and incidence of CM with age, particularly after age 50 [27], we found the highest prevalence of the disease among individuals older than 50 years.

Gender is also considered a prognostic factor for cutaneous melanoma [28]. In a study conducted in Canada during the period from 1956 to 2005, there was a significant variation in the location of the primary lesion according to gender [28]. Men tend to have tumors in the axial region, which are more likely to develop metastatic lesions [25, 29]. In our study, women presented more lesions on limbs (59.8%), while men presented lesions on the head, neck, and torso (58.9%).

### 4.5. Nevi

Several studies claim that the presence of atypical, multiple melanocytic or congenital nevi is strongly correlated to the emergence of CM. A frequency of clinically atypical nevi in patients with a history of CM, ranging from 34 to 59%, has been previously reported [30]. Data from this study showed that 9.7% of patients had atypical nevi, a much lower incidence than expected, which may be due to underdiagnosis.

There is still much controversy regarding the classification and risk of development of CM from congenital melanocytic nevi [31]. In this study, the likelihood of developing CM from a congenital nevus could not be assessed, but we found that 18.7% of patients had congenital nevi, and of these, 2.4% were giant, 36.9% medium, and 60.7% small.

Several studies have shown an association of multiple melanocytic nevi (especially above 50 nevi) with the development of CM [32]. In the present study, 11.4% of patients presented more and 20.7% presented less than 50 nevi, and therefore it was not possible to correlate the presence of multiple nevi with the risk of developing CM.

### 4.6. Histopathological Characteristics

In an investigation of clinical and histological factors associated with melanoma thickness conducted in New Zealand with 14 646 patients diagnosed in the period between 1996 and 2006, 48.1% of the patients showed SSM and 11.5% had NM [33]. Similarly, in this study, of 479 patients with CM, the most common type was SSM with 287 cases (59.9%), followed by NM with 90 (18.8%).

According to a recent work conducted in Australia, which evaluated 912 patients with primary CM diagnosed in a period of two years, tumor thickness, as described by Breslow, has proven to be the best reproducible prognostic factor. In contrast, Clark's level has shown low levels of interobserver agreement. The majority of patients presented Clark III and IV [34]. These results were similar to those found in western Santa Catarina, where most of the lesions (40.95%) were in Clark level IV and 26.43% in level III.

### 4.7. Sentinel Lymph Node

We found that in the western region of Santa Catarina, 16.4% of patients tested for SLN were positive. Similar data were obtained in a previous study in which 1327 patients with primary CM of intermediate thickness (1.2 to 3.5 mm) were followed from 1994 to 2002. Of these, 764 were tested for SLN and 16% were positive [35].

In the United States, the American Joint Committee on Cancer recommended complete lymphadenectomy as standard therapy for patients in stage III CM [36]. A study that evaluated 2203 patients, from 1999 to 2003, who were submitted to SLN biopsy and were SLN positive, showed that only 14% of patients who underwent lymphadenectomy had no positive sentinel lymph node [37]. The findings of this study are similar to the American data as only 14.5% of this group of patients had negative sentinel lymph nodes.

### 4.8. Stage

Several American and European studies have shown that the diagnosis of CM occurs mainly in early stages [38–40]. The analysis of all cases of CM diagnosed in the United States between 1985 and 1994, obtained from the National Cancer Data Base, showed that 62.6% of them were in stages 0 and I at diagnosis [41].

In developing countries, the diagnosis of CM usually occurs at later stages. In Brazil, several studies have demonstrated that approximately 50% of patients were classified as stages IV and V at the moment of diagnosis [42–44]. Our results differ from national data, since similarly to developed countries, 61.3% of the patients were in stages 0–II.

## 5. Conclusions

The incidence and prevalence of CM found in the western region of Santa Catarina are much higher than those found elsewhere in the country and are above the estimates proposed by the INCA. This fact is most likely due to the phenotypic characteristics of the population, which is composed mostly of descendants of Europeans with skin phototypes I and II. It can also be correlated to the life habits of the individuals from this region, such as continuous or intermittent sun exposure without the use of sunscreen causing burns, especially in childhood. Another hypothesis is that there is a high incidence of UV radiation in this region due to its location in southern Brazil, as is the case in the countries of Oceania, which have high UV radiation and the highest incidence of CM. The average age of CM patients was 50 years, but, considering the prevalence according to age, an increased rate of CM after the age 50 was observed. CM was more common among women. However, unfavorable prognostic factors were more related to men as well as the largest number of ulcerated lesions and the greater depth of dermal invasion. The most common histological subtypes in order of occurrence were SSM, NM, and LMM. Most lesions showed tumor thickness indices between 1 and 2 mm, Clark's levels between III and IV, and a mitotic index of ≥1/mm². In most cases, the disease was at an early stage (0, I, or II) at the moment of diagnosis.

## Figures and Tables

**Table 1:** Number of cases per year and incidence of cutaneous melanoma in the west of Santa Catarina, Brazil (*n* = 497).

Year	Number of cases of CM	Western region population*	Incidence of CM (cases/100.000 inhabitants)
2002	50	678 428	7.4
2003	55	680 118	8.1
2004	51	681 796	7.5
2005	60	685 586	8,7
2006	64	687 586	9.3
2007	60	689 568	8,7
2008	89	727 014	12.2
2009	68	731 679	9.3

*Calculation based on data from the Brazilian Institute of Geography and Statistics (2002–2009).

**Table 2:** Clinical and histopathological characteristics of patients with cutaneous melanoma in the western region of Santa Catarina, Brazil, classified by gender.

Characteristics (*n*)	Men	Women	*P*
Age (497)	50.45 ± 15.20	50.81 ± 15.57	0.045**
Site (489)			<0.001*
Head and neck	36 (17.82)	55 (19.16)	
Trunk	73 (36.14)	55 (19.16)	
Members	76 (37.62)	164 (57.14)	
Unknown	17 (8.42)	13 (4.54)	
Histological type (445)			0.04*
SSM	104 (59.10)	183 (68.03)	
NM	42 (23.86)	48 (17.84)	
LMM	13 (7.39)	20 (7.43)	
MAL	8 (4.54)	9 (3.35)	
Other	9 (5.11)	9 (3.35)	
Tumor thickness (mm) (462)			<0.01*
≤1 mm	73 (39.46)	135 (48.74)	
1.01–2.0	29 (15.67)	54 (19.49)	
2.01–4.0	28 (15.14)	49 (17.69)	
>4.0	55 (29.73)	39 (14.08)	
Clark (420)			<0.01*
I	26 (15.76)	29 (11.37)	
II	16 (9.70)	42 (16.47)	
III	33 (20)	78 (30.59)	
IV	72 (43.64)	100 (39.22)	
V	18 (10.90)	6 (2.35)	
Presence of ulceration (418)			0.013*
Yes	67 (41.61)	78 (30.35)	
No	94 (58.39)	179 (69.65)	
Presence of regression (399)			0.254*
Yes	37 (24.02)	49 (20.00)	
No	117 (75.98)	196 (80.00)	
Mitotic index (mitoses/mm^2^) (401)			0.045*
0	27 (17.65)	49 (19.76)	
≥1	126 (82.35)	199 (80.24)	
SLN positive (225)			0.600*
Yes	21 (24.70)	16 (11.42)	
No	64 (72.30)	124 (88.57)	
Growth phase (434)			0.271*
Radial	28 (16.57)	49 (18.49)	
Vertical	141 (83.43)	216 (81.51)	
Vascular invasion (400)			0.877*
Yes	16 (10.19)	24 (9.88)	
No	141 (89.81)	219 (90.12)	
Perineural invasion (392)			0.398*
Yes	6 (3.82)	4 (1.70)	
No	151 (96.18)	231 (98.30)	

Data presented as mean ± SD and frequency (percentage). **Student's* t-test* * *Chi*-square test. SSM, superficial spreading melanoma; NM, nodular melanoma; LMM, lentigo maligna melanoma; MAL, acrolentiginous melanoma; SLN, sentinel lymph node.

## References

[1] Gilchrest BA, Eller MS, Geller AC, Yaar M (1999). The pathogenesis of melanoma induced by ultraviolet radiation. *The New England Journal of Medicine*.

[2] Eide MJ, Weinstock MA (2005). Association of UV index, latitude, and melanoma incidence in nonwhite populations. *Archives of Dermatology*.

[3] Pearce J, Barnett R, Kingham S (2006). Slip! Slap! Slop! Cutaneous malignant melanoma incidence and social status in New Zealand, 1995–2000. *Health and Place*.

[4] Parkin M, Bray F, Ferlay J, Pisani P (2005). Global cancer statistics, 2002. *Ca-A Cancer Journal for Clinicians*.

[5] Lens MB, Dawes M (2004). Global perspectives of contemporary epidemiological trends of cutaneous malignant melanoma. *British Journal of Dermatology*.

[6] Salmon PJM, Chan WC, Griffin J, McKenzie R, Rademaker M (2007). Extremely high levels of melanoma in Tauranga, New Zealand: possible causes and comparisons with Australia and the northern hemisphere. *Australasian Journal of Dermatology*.

[7] Machado AT, Oliveira BR, Charles A (2004). Conduta para o melanoma cutâneo maligno. *Revista Médica de Minas Gerais*.

[8] Instituto Nacional do Câncer (INCA) http://www.inca.gov.br/estimativa/2012/index.asp?link=conteudo_view.asp&ID=5.

[9] AUSTRALIAN GOVERNMENT http://www.australia.gov.au/.

[10] INSTITUTO BRASILEIRO DE GEOGRAFIA E BIOESTATÍSTICA (IBGE) http://www.mapas.ibge.gov.br/.

[11] http://www.gbm.org.br/.

[12] DATASUS Banco de Dados do Sistema Único de Saúde.

[13] Marks R (1996). Prevention and control of melanoma: the public health approach. *Ca-A Cancer Journal for Clinicians*.

[14] Ferrari NM (2006). *Estudo Epidemiológico Descritivo dos Doentes de Melanoma Cutâneo Acompanhados na Unidade de Melanoma da Santa Casa de São Paulo*.

[15] Balch CM, Soong SJ, Atkins MB (2004). An evidence-based staging system for cutaneous melanoma. *Ca-A Cancer Journal for Clinicians*.

[16] Lancaster HO (1956). Some geographical aspects of the mortality from melanoma in Europeans. *The Medical Journal of Australia*.

[17] Holman CDJ, Armstrong BK (1987). Malignant melanoma of the skin. *Bulletin of the World Health Organization*.

[18] Boniol M, Vries ED, Coebergh JW, Doré JF (2005). Seasonal variation in the occurrence of cutaneous melanoma in Europe: influence of latitude. An analysis using the EUROCARE group of registries. *European Journal of Cancer*.

[19] Bakos L, Masiero NC, Burttet RM (2010). Is season important for the diagnosis of cutaneous melanoma in southern Brazil? A 10-year hospital-based study. *International Journal of Dermatology*.

[20] Ammirati CT, Hruza GJ (2005). Clinical presentations of cutaneous melanoma. *Facial Plastic Surgery Clinics of North America*.

[21] Parker SL, Tong T, Bolden S, Wingo PA (1996). Cancer statistics. *Ca-A Cancer Journal for Clinicians*.

[22] Burton RC (2000). Malignant melanoma in the year 2000. *Ca-A Cancer Journal for Clinicians*.

[23] Coory M, Smithers M, Aitken J, Baade P, Ring I (2006). Urban-rural differences in survival from cutaneous melanoma in Queensland. *Australian and New Zealand Journal of Public Health*.

[24] Instituto Nacional de Pesquisas Espaciais (INPE) http://www.satelite.cptec.inpe.br/uv/.

[25] Scoggins CR, Ross MI, Reintgen DS (2006). Gender-related differences in outcome for melanoma patients. *Annals of Surgery*.

[26] Gomez BP, Aragones N, Gustavsson P, Lope V, López-Abente G, Pollán M (2008). Socio-economic class, rurality and risk of cutaneous melanoma by site and gender in Sweden. *BMC Public Health*.

[27] Caracò C, Marone U, Botti G, Celentano E, Lastoria S, Mozzillo N (2006). Age as predictor in patients with cutaneous melanoma submitted to sentinel lymph node biopsy. *European Journal of Surgical Oncology*.

[28] Pruthi DK, Guilfoyle R, Nugent Z, Wiseman MC, Demers AA (2009). Incidence and anatomic presentation of cutaneous malignant melanoma in central Canada during a 50-year period: 1956 to 2005. *Journal of the American Academy of Dermatology*.

[29] Rampen F (1980). Malignant melanoma: sex differences in survival after evidence of distant metastasis. *British Journal of Cancer*.

[30] Naeyaert JM, Brochez L (2004). Dysplastic nevi. *The New England Journal of Medicine*.

[31] Fernandes NC, Machado JLR (2009). Clinical study of the congenital melanocytic naevi in the child and adolescent. *Anais Brasileiros de Dermatologia*.

[32] Gandini S, Sera F, Cattaruzza MS (2005). Meta-analysis of risk factors for cutaneous melanoma: III. Family history, actinic damage and phenotypic factors. *European Journal of Cancer*.

[33] Sneyd MJ, Cox B (2011). Clinical and histologic factors associated with melanoma thickness in New Zealand Europeans, Maori, and Pacific peoples. *Cancer*.

[34] Murali R, Hughes MT, Fitzgerald P, Thompson JF, Scolyer RA (2009). Interobserver variation in the histopathologic reporting of key prognostic parameters, particularly clark level, affects pathologic staging of primary cutaneous melanoma. *Annals of Surgery*.

[35] Morton DL, Thompson JF, Cochran AJ (2006). Sentinel-node biopsy or nodal observation in melanoma. *The New England Journal of Medicine*.

[36] Spillane AJ, Winstanley J, Thompson JF (2009). Lymph node ratio in melanoma: a marker of variation in surgical quality?. *Cancer*.

[37] Gershenwald JE, Andtbacka RHI, Prieto VG (2008). Microscopic tumor burden in sentinel lymph nodes predicts synchronous nonsentinel lymph node involvement in patients with melanoma. *Journal of Clinical Oncology*.

[38] Vries E, Bray FI, Coebergh JWW, Parkin DM (2003). Changing epidemiology of malignant cutaneous melanoma in Europe 1953–1997: rising trends in incidence and mortality but recent stabilizations in western Europe and decreases in Scandinavia. *International Journal of Cancer*.

[39] Hall HI, Miller DR, Rogers JD, Bewerse B (1999). Update on the incidence and mortality from melanoma in the United States. *Journal of the American Academy of Dermatology*.

[40] Balch CM, Soong SJ, Gershenwald JE (2001). Prognostic factors analysis of 17,600 melanoma patients: validation of the American joint committee on cancer melanoma staging system. *Journal of Clinical Oncology*.

[41] Chang AE, Karnell LH, Menck HR (1998). The national cancer data base report on cutaneous and noncutaneous melanoma: a summary of 84,836 cases from the past decade. *Cancer*.

[42] Proença NG, Bernardes MF, Muller HJ (1991). Fatores que influenciam sobre o prognóstico do melanoma maligno. *Anais Brasileiros de Dermatologia*.

[43] Criado PR, Vasconcellos C, Sittart JA (1999). Primary cutaneous malignant melanoma: retrospective studyfrom 1963 to 1997 at Hospital do servidor público estadual de são Paulo. *Revista da Associação Médica Brasileira*.

[44] Gon AS, Minelli L, Guembarovski AL (2001). Primary cutaneous melanoma in londrina. *Anais Brasileiros de Dermatologia*.

